# Marinopyrrole derivative MP1 as a novel anti-cancer agent in group 3 MYC-amplified Medulloblastoma

**DOI:** 10.1186/s13046-024-02944-w

**Published:** 2024-01-11

**Authors:** Don W. Coulter, Yashpal S. Chhonker, Devendra Kumar, Varun Kesherwani, Wafaa N. Aldhafiri, Erin M. McIntyre, Gracey Alexander, Sutapa Ray, Shantaram S. Joshi, Rongshi Li, Daryl J. Murry, Nagendra K. Chaturvedi

**Affiliations:** 1 Department of Pediatrics, Hematology/Oncology Division, University of Nebraska Medical Center, Omaha, NE 68198 USA; 2 Department of Pharmacy Practice & Science, University of Nebraska Medical Center, Omaha, NE 68198 USA; 3 Child Health Research Institute, University of Nebraska Medical Center, Omaha, NE 68198 USA; 4 Department of Genetics, Cell Biology and Anatomy, University of Nebraska Medical Center, Omaha, NE 68198 USA; 5 Fred & Pamela Buffett Cancer Center, University of Nebraska Medical Center, Omaha, NE 68198 USA; 6 Department of Pediatrics, Hematology and Oncology Division, University of Nebraska Medical Center, Omaha, NE 68198 USA

**Keywords:** Medulloblastoma, Marinopyrroles, MP1, MYC, mTOR/translation, Metabolism

## Abstract

**Background:**

Medulloblastoma (MB) patients with MYC oncogene amplification or overexpression exhibit extremely poor prognoses and therapy resistance. However, MYC itself has been one of the most challenging targets for cancer treatment. Here, we identify a novel marinopyrrole natural derivative, MP1, that shows desirable anti-MYC and anti-cancer activities in MB.

**Methods:**

In this study, using MYC-amplified (Group 3) and non-MYC amplified MB cell lines in vitro and in vivo, we evaluated anti-cancer efficacies and molecular mechanism(s) of MP1.

**Results:**

MP1 significantly suppressed MB cell growth and sphere counts and induced G2 cell cycle arrest and apoptosis in a MYC-dependent manner. Mechanistically, MP1 strongly downregulated the expression of MYC protein. Our results with RNA-seq revealed that MP1 significantly modulated global gene expression and inhibited MYC-associated transcriptional targets including translation/mTOR targets. In addition, MP1 inhibited MYC-target metabolism, leading to declined energy levels. The combination of MP1 with an FDA-approved mTOR inhibitor temsirolimus synergistically inhibited MB cell growth/survival by downregulating the expression of MYC and mTOR signaling components. Our results further showed that as single agents, both MP1 and temsirolimus, were able to significantly inhibit tumor growth and MYC expression in subcutaneously or orthotopically MYC-amplified MB bearing mice. In combination, there were further anti-MB effects on the tumor growth and MYC expression in mice.

**Conclusion:**

These preclinical findings highlight the promise of marinopyrrole MP1 as a novel MYC inhibition approach for MYC-amplified MB.

**Supplementary Information:**

The online version contains supplementary material available at 10.1186/s13046-024-02944-w.

## Background

Medulloblastoma (MB) is the most common intracerebellar pediatric brain tumor, accounting for approximately 20% of all childhood brain tumors and over 60% of embryonal brain tumors. Standard-of-care treatment for MB consists of surgery, radiation, and chemotherapy, and approximately one-third of children die from this disease. Moreover, severe treatment-related toxicity remains a major problem for survivors [1, 2]. Extensive genetic, epigenetic, and transcriptomic analyses have identified MB as a heterogenous disease with four major molecular subgroups namely wingless (WNT pathway-activated), sonic-hedgehog (SHH pathway-activated) Group 3 and Group 4 [3–7]. Of these, Group 3 MB often exhibits MYC amplification (17–20% of cases) or overexpression, metastasis (40–50% of cases), and treatment failure, and it has the worst prognosis of the MB subgroups with < 60% survival [5, 8, 9]. Thus, there is an urgent and unmet need to develop new targeted therapies for the treatment of such MB while acquiring limited toxicities.

Molecularly targeted therapies are better tolerated than traditional therapies and have prolonged patient survival [10]. Targeting MYC has high potential therapeutic value due to its wide role in cancer development, its overexpression in varied cancers (approximately 70% of cancers), and its association with a poor prognosis [11]. Currently, there are no effective small-molecule therapeutic agents available for treating MYC-amplified MB. In the past, drug discovery approaches aimed at blocking MYC heterodimerization with MAX or its binding with DNA have largely failed [11, 12]. While alternative indirect strategies have been proposed, due to the broad role and the number of pathways affected by its overexpression, MYC remains challenging to target.

Marinopyrroles are novel alkaloids isolated from a marine Streptomyces [13]. These compounds were initially developed as a new class of antibacterials but subsequently were reported to have anti-cancer activities based on their ability to inhibit MCL-1 expression [14–16]. MCL-1 is an anti-apoptotic protein, highly expressed in a variety of human cancers and a validated drug target for cancer treatment [16]. We previously reported marinopyrroles as active antibiotic and anticancer agents [17, 18]. To further improve physicochemical and drug-like properties, we designed a novel series of pyrrolomycin-based natural derivatives. Our previous studies have demonstrated that a novel natural product derivative, MP1 (Marinopyrrole/Pyrrolomycin-1), has desirable physicochemical and drug-like properties, potent anti-tumor activity in MYCN-amplified neuroblastoma cell lines (IC_50_ = 96 nM), an acceptable pharmacokinetic profile in mice, and significant antitumor efficacy in vivo against MYCN-driven neuroblastoma when used in combination with temsirolimus, an FDA approved mTOR inhibitor [19, 20]. In particular, MP1 is able to inhibit protein expression of MYCN (a MYC homologue) and suppress MYCN-driven tumorigenic programs in neuroblastoma cells. Importantly, it is evident from our biodistribution study that MP1 can cross the blood–brain-barrier [20].

These findings prompted us to explore if MP1 has anticancer efficacy against MB and the ability to target MYC protein and its associated oncogenic programs. This study expands the pre-clinical validation of MP1 as a promising lead compound that could hold great potential as a therapy for MB. MYC amplification or overexpression drives several pathways involved in cancer development and drug-resistance [12]. One of the major pathways regulated by MYC is the protein synthesis (translation)/mTOR signaling pathway. This association between MYC and mTOR signaling, along with the current use of the mTOR inhibitor temsirolimus in clinical trials, further prompted us to explore anti-MB efficacies of MP1 in combination with temsirolimus.

## Methods

### Cell lines and their maintenance

Daoy (non-MYC-amplified), D-283 (MYC-overexpressed) and D-341 (MYC-amplified) MB cell lines were purchased from American Type Culture Collection. HD-MB03 (MYC-amplified) MB cell line was purchased from Deutsche Sammlung von Mikroorganismen und Zellkulturen (Germany). ONS-76 (non-MYC-amplified, SHH) MB cell line was purchased from Sekisui-XenoTech (USA). PDX MB cell lines MED-114FH (Group 3, MYC-amplified) and MED-411-FH (Group 3 MB, MYC-amplified) were obtained from the Brain Tumor Resource Lab (BTRL), Seattle Children’s Hospital. ONS-76 RFP (control vector) and ONS-76 MYC-OE (MYC-overexpressed) cell lines were obtained from Dr. Rajeev Vibhakar (University of Colorado, Aurora, CO).

These cell lines were authenticated by their respective resources using short tandem repeat profiling. All cell lines were also tested for negative mycoplasma contamination using the MycoSensor-PCR assay kit (Agilent-Technologies). The established cell lines (Daoy, ONS-76, D-283, D-341, HD-MB03) were cultured and maintained using RPMI-1640 media supplemented with 10% heat-inactivated FBS and 1% penicillin–streptomycin in a humidified incubator at 5% CO_2_ and 95% air atmosphere at 37 °C. The PDX cell lines (MED-114FH, MED-411FH) were cultured as spheroids in non-coated plates using NeuroCult NS-A media supplemented with proliferation supplements (Stem Cell Technologies) and 1% penicillin–streptomycin in a humidified incubator at 5% CO_2_ and 95% air atmosphere at 37 °C. Experiments were performed using fewer than 10 passages for each cell line.

### Anticancer agents (MP1 and temsirolimus)

MP1 (purity: ≥ 98%) was synthesized by Dr. Rongshi Li, a co-author in this manuscript. The toxicity, pharmacokinetic (PK), and brain-penetrance properties of this compound have been reported by us [19, 20]. Temsirolimus was purchased from MedChemExpress LLC. These inhibitors were dissolved in dimethyl-sulfoxide (DMSO) at 1- or 10-mM concentrations and stored at -20 °C. In each experiment, an equivalent amount of DMSO for the highest drug concentration was used as solvent control.

### Cell growth, apoptosis, and cell cycle analyses

Cell growth, apoptosis, and cell cycle analyses in MB cells treated with inhibitors were performed using the MTT assay, Annexin-V assay and propidium-iodide staining, respectively, as described previously [21–23].

### Western blotting

Western blot analysis was performed using a previously described protocol in our lab [22]. Primary antibodies used in this analysis included MYC (#18583), MCL-1 (#94296), p-4EBP1-ser65 (#9456), 4EBP1 (#9452), p-EIF4E-ser209 (#9741), EIF4E (#9742), p-S6K-thr421/ser424 (#9204), S6K (#9202), cyclophilin-B (#43603), p21 (#2947), GAPDH (#5174), Vinculin (#13901), Cleaved-caspase-3 (#9661), CD133 (#64326), and Cyclin-B1 (#12231). All these antibodies were purchased from Cell Signaling Technology.

### Quantitative RT-PCR (qRT-PCR)

RNA was prepared using the RNeasy kit (Qiagen) and 2 µg of total RNA was used for cDNA preparation using Superscript Verso enzyme kit (Promega). cDNA product was amplified in a 10 µl reaction using SYBR-Green Supermix and standard gene-specific (MYC, CD133, GAPDH) primers (Applied Biosystems). All reactions were processed in a QuantStudio-3 PCR System and results analyzed by QuantStudio software (Applied Biosystems).

### Cycloheximide chase experiment

To determine the stability of MYC protein, MYC-amplified (HD-MB03) cells were treated with 50 μg/ml cycloheximide (Sigma Aldrich) following MP1 treatment for 24 h. Following treatment, protein lysates from the indicated time points of cycloheximide treatments were subjected to western blotting for MYC protein.

### Sphere assay

Twenty thousand MB (MED-114FH, MED-411FH) cells resuspended in NeuroCult medium were seeded in non-coated 6-well plates and allowed 72 h to form spheres. Spheres were treated with MP1 for an additional 72 h. Following treatment, aggregates of cells > 50 µm in diameter were counted and imaged using an EVOS-Auto-Imaging System (Life Technologies). Spheres were also subjected to western blot analysis for the expression of MYC protein.

### Protein synthesis assay

MB cells were treated with MP1 in 96-well plates (2 × 10^4^ cells/well) for 24 h. After treatment, culture media was replaced with fresh media containing O-propargyl-puromycin (OPP) and incubated for 30 min at 37 °C to be incorporated in translating polypeptide chains. Cells were then fluorescently stained with 5-FAM‐Azide. The detection of fluorescent-labelled OPP was performed using the Protein Synthesis Assay Kit (#601100, Cayman Chemical, USA), according to the manufacturer’s instructions.

### Metabolome analysis

HD-MB03 cells were cultured in RPMI media containing vehicle or MP1 (0.25 µM) for 24 h. Following incubation, cells were rinsed and extracted with 80% ice-cold methanol on dry ice for polar metabolites. Targeted metabolomic data analysis was performed as described previously [24].

### Seahorse analyses

The extracellular acidification rate (ECAR) and oxygen consumption rate (OCR) of the cells was assessed using the Seahorse XFp Flux Analyzer (Seahorse Bioscience, Agilent) according to the manufacturer’s instructions. In brief, 2 × 10^4^ HD-MB03 cells were seeded in a Seahorse XFp 8-well assay plate and incubated at 37 °C overnight for adhesion. Cells were then treated with vehicle (control) and 0.25 µM MP1 for 24 h. Before detection, culture medium was replaced with assay media (10 mM glucose, 1 mM sodium pyruvate and 2 mM glutamine, PH 7.4) and incubated at 37 °C for 1 h. Basal levels of ECAR and OCR were recorded over 24 min, followed by glycolytic and mitochondrial stress tests. The results were graphed on a grid indicating degree of aerobic/glycolytic metabolic activities.

### RNA sequencing and gene expression analyses

RNA from MP1-treated HD-MB03 cells, was purified using the Qiagen RNeasy Kit. After confirming sequence grade quality of RNA using an Agilent 2100 Bioanalyzer, an RNA library was prepared using True-Seq RNA Sample Prep V2 Kit and subjected to RNA sequencing using the Illumina NextSeq550 system in the UNMC Genomics Core Facility. Each sample was processed in triplicate. The original fastq reads were processed by a newly developed standard pipeline utilizing STAR as the aligner and RSEM as the tool for annotation and quantification at both gene and isoform levels. Using these reads, the normalized FPKM and TPM values for all the available genes were calculated and then differential gene expression and gene-set-enrichment analysis (GSEA) between treatment groups were performed. Differential gene expression, pathway and gene-set-enrichment (GSE) analyses between treatment groups were performed by TACGenomics at the UCLA genomic center.

### Animal studies

All animal experiments were performed according to a UNMC Institutional Animal Care and Use Committee (IACUC) approved protocol. For subcutaneous xenograft study, six- to eight-week-old NSG female mice from Jackson Laboratories were subcutaneously injected in the right-flank with 2.5 × 10^5^ HD-MB03 MB cells suspended in 100 µl PBS and mixed 1:3 with matrigel. Ten days post-tumor injection, when tumor was palpable, the tumor bearing mice were divided into four treatment groups (*n* = 5 each group) and underwent treatments for three weeks. The vehicle control treatment was (5% DMSO + 45% PEG 300 + 2% Tween 80. Experimental treatments were MP1 (10 mg/kg, p.o. 5 × per week), temsirolimus (10 mg/kg, i.p. 3 × per week), or the combination of MP1 with temsirolimus. Tumor volume was assessed twice a week using the digital caliper. When tumor volume approached 2 cm^3^, the mice were euthanized using CO_2_ and tumor tissues and vital organs were collected and processed for histological and immunohistological analyses. Immunohistochemical (IHC) analysis was performed using a previously described protocol in our lab [21]. Primary antibodies used in this analysis included MYC (1:500 dilution) and Ki-67 (1:500 dilution) rabbit antibodies. These IHC grade antibodies were purchased from Abcam.

For the orthotopic xenografts study, 1 × 10^5^ HD-MB03 cells suspended in 5 µl PBS were injected into cerebella of NSG mice using the digital stereotaxic instrument (Stoelting Instrument) and guidance. Ten days following injection, mice were randomly divided into four treatment (vehicle, MP1, TEM and MP1 + TEM) groups (each group *n* = 5) and treated with inhibitors as indicated above for subcutaneous xenografts. Survival of mice was evaluated by daily monitoring of severe ataxia, tumor-related morbidity, and weight loss > 20% as endpoints. In addition, cerebellar tumors were examined by histological (H&E) analysis.

### Statistical analysis

All experiments were repeated at least three times and the mean and standard error values calculated. Statistically significant differences were calculated using Student t-tests or analysis of variance (ANOVA) and a significance threshold of *p* < 0.05 or *p* < 0.01, as noted. The data analyses, including IC_50_ calculation, were carried out using Microsoft Excel and GraphPad Prism 7.0 (San Diego, CA, USA) software. To determine synergy, we employed the Chou and Talalay CI method using CalcuSyn software (Biosoft, UK). CI < 0.9 indicates synergism, 0.9–1.1 additivity and > 1.1 antagonism.

## Results

### Growth inhibitory efficacy of MP1 against MB cells

**Fig. 1:**
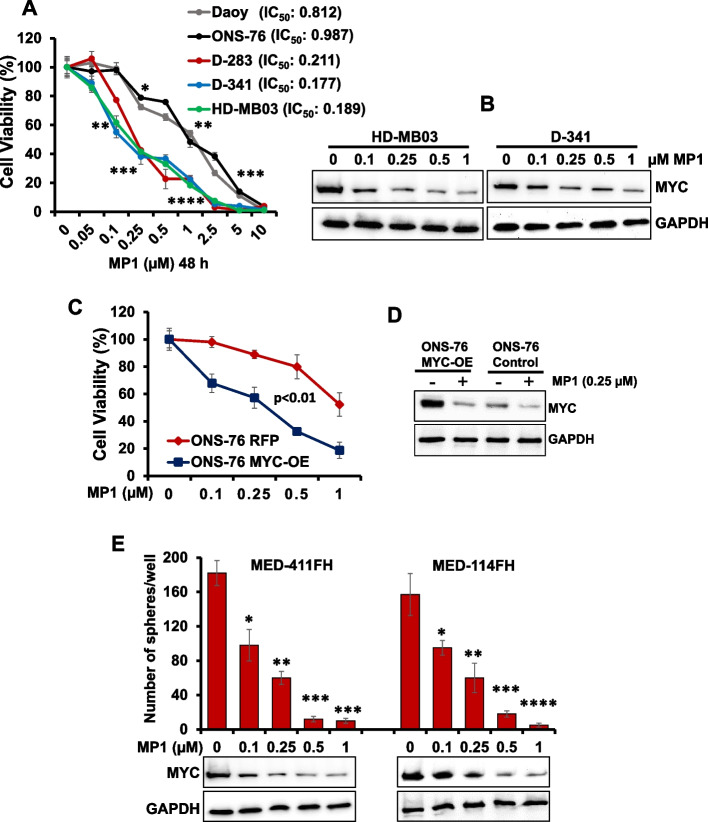
Inhibitory effects of MP1 on MB cell growth and MYC protein expression. **A** MTT (cell growth) assay showing dose-dependent growth effects of MP1 with the low-µM doses as indicated, in MB cell lines (with corresponding IC_50_) at 48 h. Percentage of viable cells is relative to DMSO- (solvent control)-treated cells. Plotted values and error bars represent mean ± SEM. **B** Western blot results show dose-dependent effect of MP1 on MYC protein expression in MYC-amplified MB cell lines at 24 h. **C** MTT assay showing the sensitization of MYC overexpressed ONS-76 (ONS-76 MYC-OE) cells to MP1 (48 h) treatment compared to the control ONs-76 RFP cells. Plotted values and error bars represent mean ± SEM. Control ONS-76 vs ONS-76 MYC-OE; *p* < 0.01 (Student-t-test). **D** MYC protein expression in MYC overexpressed ONS-76 and ONS-76 control cells treated with 0.25 µM MP1 for 24 h. **E** Quantification of the number of spheres following dose-dependent treatment of MP1 in two PDX-derived MB cell lines (MED-411FH, MED-114FH) at 72 h. Values, mean ± SEM. *p < 0.05; **p < 0.01; ***p < 0.005; ****p < 0.001 (Student-*t*-test). Below are western blot results showing corresponding changes in MYC protein expression after MP1 treatment. GAPDH was used as the loading control in all western blot experiments presented in this figure

To examine the growth inhibitory efficacy of MP1 against MB cells in vitro, the two non-MYC-amplified (Daoy, ONS-76) and three MYC-amplified/overexpressed MB cell lines (D-341, HD-MB03, D-283) were treated with MP1 (0.05–10 μM) in a dose-dependent manner for 48 h, and the growth/viability of the cells was assessed using MTT assay. The concentrations used for MP1 were chosen from our previous published studies [19, 20]. The MTT result shown in Fig. 1A clearly showed a dose-dependent growth inhibition of all MB lines following treatment with MP1. MP1 was able to inhibit growth of MYC-amplified MB cell lines with lower IC_50_ (0.17 to 0.21 μM) and showed efficacy against non-MYC-amplified MB cell lines with approximately fourfold higher IC_50_ (0.81 to 0.98 μM), suggesting higher sensitivity of MYC-amplified MB cells to MP1. Notably, the growth inhibition of MYC-amplified MB cell lines by MP1 was correlated with reduced MYC protein expression, in a dose-dependent manner (Fig. 1B, Supplementary Fig. S1A). The IC_50_ values of MP1 in MYC-amplified MB cell lines were within achievable range of the concentrations observed in the plasma and brain of mice in our previous studies [20]. Overall, these results demonstrated the anti-cancer potential of MP1 against MYC-amplified MB cells.

To further show the MYC-dependent efficacy of MP1 in MB, we used ONS-76 cells, a low MYC-expressing MB cell line, with an exogenous MYC overexpression lentivirus vector. Control vector and MYC-overexpressing ONS-76 cells were treated with increasing concentrations of MP1 for 48 h, and growth/viability was assessed. Our data revealed that MYC-overexpressing ONS-76 cells treated with MP1 showed greater cell growth inhibition, compared to control ONS-76 cells (Fig. 1C), suggesting MYC overexpression increases MB cells sensitivity to MP1. The growth inhibition of these cells by MP1 was associated with reduced MYC expression (Fig. 1D, Supplementary Fig. S1B).

We also confirmed the efficacy of MP1 using two MYC-amplified Group 3 MB (Med-114FH, Med-411FH) PDX cell lines characterized previously [25]. Since these PDX lines grow as sphere forms, we tested MP1 efficacy on the spheres of these lines. The established MB spheres (in Neuro-cult culture media) were treated with increasing concentrations of MP1 followed by the assessment of sphere numbers and MYC expression. Results showed a dose-dependent efficacy of MP1 on inhibiting sphere counts/formation and MYC protein expression in both PDX cell lines (Fig. 1E, Supplementary Fig. S1C), suggesting anti-cancer efficacy of MP1 on MB spheres.

### Induction of cell cycle arrest and apoptosis by MP1 in MB cells

**Fig. 2:**
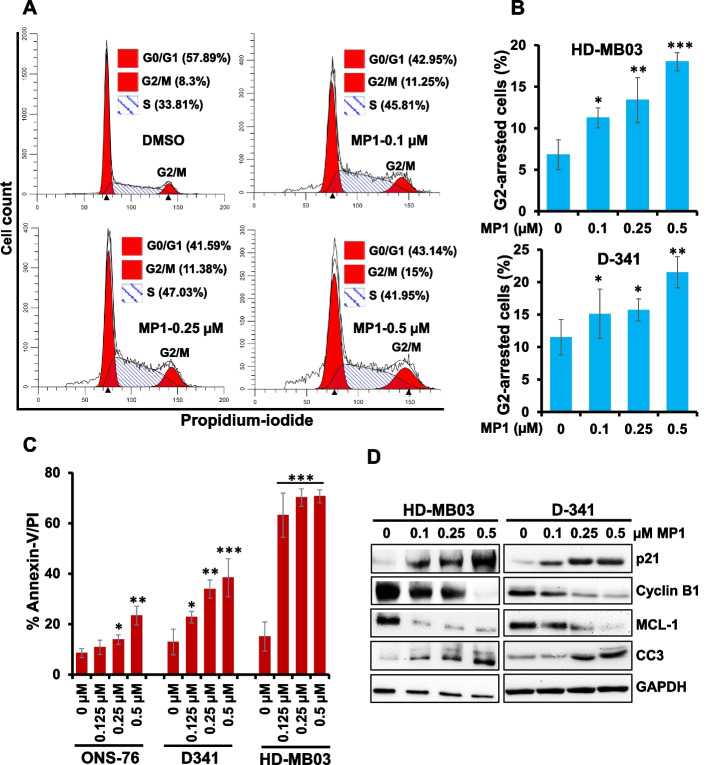
MP1 arrests G2-cell cycle and induces apoptosis. **A** Representative flow diagrams for cell cycle distribution of MYC-amplified MB cell line HD-MB03 following dose-dependent treatment with MP1 for 24 h and then staining with propidium-iodide (PI). **B** Quantification of the percentage of G2-phase arrested cells following treatment of two MYC-amplified (HD-MB03, D-341) cell lines with MP1 across a dose range for 24 h. The results shown here reflect three replicates. Values, mean ± SEM. *p < 0.05; **p < 0.01; ***p < 0.005 (Student-*t*-test). This *p*-value denotes significance between 0 (DMSO solvent) and each MP1 treatment. **C** Bar graphs show flow cytometry-derived quantification of Annexin-V/PI double positive apoptotic cells in two MYC-amplified (HD-MB03, D-341) and one non-MYC-amplified (ONS-76) MB cell lines treated with MP1 as indicated. Results are representative of three independent experiments. Plotted values and error bars represent mean ± SEM. *p < 0.05; **p < 0.01; ****p < 0.001 (Student-*t*-test). The p-values denote significance between 0 (DMSO-control) and each MP1 treatment. **D** Western blotting for the expression of proteins associated with G2 phase of the cell cycle (Cyclin B1, p21) and apoptosis (MCL-1, Cleaved-caspase 3) in MP1 treated HD-MB03 and D-341 cells (as indicated) for 24 h. Vinculin was used as the loading control in this experiment

To determine the efficacy of MP1 to induce cell cycle arrest and apoptosis, MB cell lines were treated with MP1 at sub-IC_50_ or IC_50_ concentrations (observed in MYC-amplified MB cell lines in Fig. 1) for 48 h and subjected to cell cycle analysis using propidium-iodide staining and apoptosis analysis using Annexin-V staining. The cell cycle profile (Fig. 2A and B) in MYC-amplified (D-341, HD-MB03) cell lines showed that MP1 arrested the cells in G2 phase of the cell cycle in a concentration-dependent manner. Results of our apoptosis analysis using the Annexin-V assay clearly showed that MP1 significantly induced apoptosis in MB cell lines in a dose-dependent manner with a greater efficacy in MYC-amplified MB cell lines, compared to non-MYC-amplified ONS-76 cells (Fig. 2C). As expected, MP1 treatment strongly induced the expression of p21 (G2 cell cycle inhibitor protein) and cleaved caspase-3 (apoptosis marker), while inhibiting the expression of Cyclin-B1 (G2 cell cycle inducer) and MCL-1 (anti-apoptotic protein) in MYC-amplified MB cells (Fig. 2D, Supplementary Fig. S1D). Together, these results suggest that MP1 suppresses the cell growth of MYC-amplified MB cells by inducing G2 cell cycle arrest and apoptosis.

#### MP1 suppresses MYC-induced transcriptional programs in MYC-driven MB cells

**Fig. 3:**
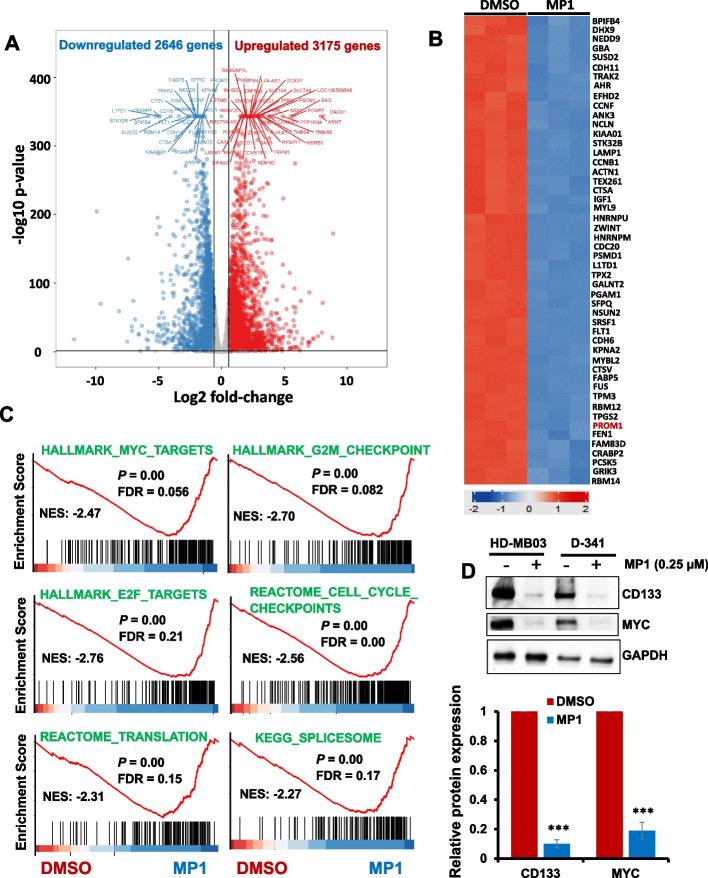
Effects of MP1 on global gene expression. Using RNA-seq, gene expression studies were performed in HD-MB03 cells 24 h after treatment with DMSO (vehicle control) or MP1 (0.25 µM). **A** Volcano plot showing total number of genes significantly upregulated or downregulated by MP1, compared to DMSO. **B** Heatmap showing the top 50 genes most significantly downregulated by MP1 treatment. **C** GSE analysis for the enrichment/modulation of cancer target gene sets (including MYC-associated target gene sets) by MP1, compared to DMSO. FDR, false discovery rate. **D** Western blotting for the expression of CD133 (one of the top 50 downregulated genes by MP1) along with MYC protein in HD-MB03 and D-341 cell lines treated with 0.25 µM MP1 for 24 h. GAPDH was used as the loading control in this experiment. Bar graphs show the quantification of expression of key target proteins (shown in western blot images) relative to the DMSO control in the combined blots of HD-MB03 and D-341 cells after GAPDH (loading control) normalization using Image-J software. The values represent the mean ± SEM of three blot replicates. ***p < 0.001 (Student t test, DMSO vs. MP1)

As an aberrant global gene expression caused by MYC protein plays key roles in MB tumorigenesis [26], we next wanted to mechanistically investigate the effect of MP1 on the overall transcriptional modulation of MYC-amplified MB cells. We performed RNA-sequencing in HD-MB03 cells treated with MP1 (0.25 µM) for 24 h. Using log_2_ fold-change with *p*-value < 0.001 and FDR-value < 0.01 as the cutoff, our gene expression data showed that a total of 3175 and 2646 genes (of all 13,805 detectable genes) were up- and downregulated, respectively, after MP1 treatment (Fig. 3A), suggesting MP1 efficacy to modulate MYC-driven genome-wide transcription. The top 50 differentially downregulated genes are depicted in the heatmap (Fig. 3B). An unbiased screen of pathways altered by MP1 (using Gene Set Enrichment Analysis) identified significant alteration of 30 pathways (*P* < 0.05; Supplementary Fig. S2). Of these, a significant number of cancer-related pathways, particularly MYC-target pathways, were downregulated by MP1. These downregulated pathways included MYC-target signatures, translation initiation/mTOR signaling, mRNA splicing, and cell cycle (cell cycle checkpoints, G2-M checkpoints, E2F-targets, DNA-replication) (Fig. 3C). The enrichment of these MYC-associated pathways was also found among top pathways identified by other independent (Gene Ontology, and KEGG/Reactome) pathway analyses (Supplementary Fig. S2 and S3). The modulation of these pathways, particularly translation (known to increase proliferation) and cell cycle (G2-M) pathways, agrees with our prior results showing that MP1 suppressed MB cell growth/proliferation and arrested G2-M cell cycle.

Among the genes most significantly downregulated by MP1 was the neural-stem cell oncogene *PROM1* (also known as CD133) (Fig. 3B, Supplementary Table S1). The high expression of CD133 has recently been shown to positively correlate with MYC expression and associate with relapse and poor prognosis in MB patients [27]. Using RT-PCR and western blotting in HD-MB03 and D-341 cell lines, we confirmed that MP1 significantly downregulated CD133 mRNA and protein expression in both cell lines (Fig. 3D, supplementary Fig. S4). The expression of MYC mRNA was not affected by MP1 as MYC itself is known to negatively autoregulate its mRNA expression. In contrast, MP1 strongly inhibited MYC protein expression in MB cell lines. We further tested if MP1 inhibited the expression of MYC protein by regulating its stability. We performed a cycloheximide (CHX) chase experiment in MP1-treated HD-MB03 cells and measured the half-life of MYC protein. As shown in Supplementary Fig S5, MYC has a half-life of approximately 50 min after CHX treatment in cells treated with control DMSO, whereas MP1 treatment dramatically decreased its half-life to approximately 20 min. There was approximately 30 min earlier degradation of MYC protein in MP1-treated cells, compared to control solvent-treated cells, suggesting that MP1 inhibits MYC protein expression by destabilizing it. Together, these results confirm that MP1 downregulates MYC and MYC activated or associated pathways in MB.

#### MP1 downregulates translation/mTOR pathway in MYC-driven MB cells

**Fig. 4:**
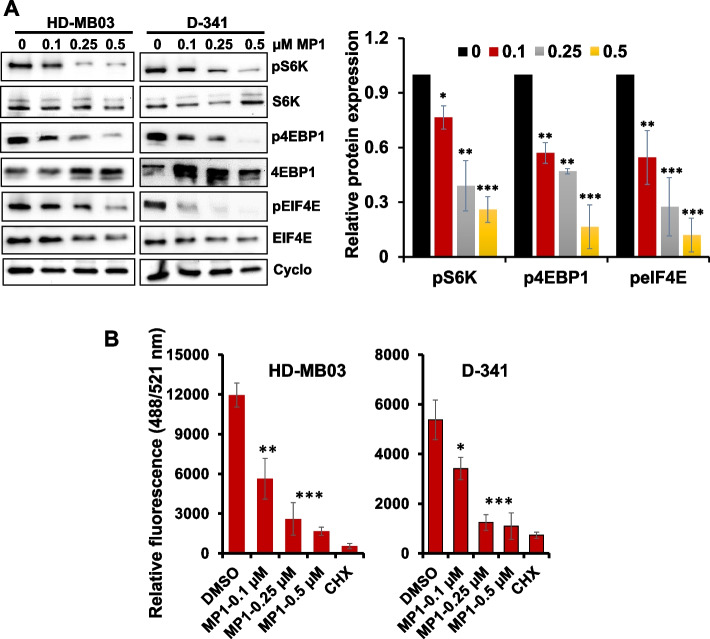
Effects of MP1 on translation pathway. **A** Western blot images for the expression of translation/mTOR components signaling in two MYC-amplified MB cell lines following treatment with MP1 in a dose-dependent manner for 24 h. Cyclophillin B was used as the loading control in these experiments. Bar graphs show the quantification of expression of key target proteins (shown in western blot images) relative to the DMSO (“0” treatment) control in the combined blots of HD-MB03 and D-341 cells after Cyclophillin B (loading control) normalization using Image-J software. The values represent the mean ± SEM of three blot replicates. The values represent the mean ± SEM of three blot replicates. *p < 0.05; **p < 0.01; ***p < 0.001 (Student t test, DMSO vs. MP1 treatments). **B** Overall protein synthesis measurement by OPP-incorporation following treatment with MP1 for 24 h. CHX (50 μg/ml, 1 h) was used as a positive control for protein synthesis inhibition. Values represent mean ± SEM. *p < 0.05; **p < 0.01; ***p < 0.005 (Student-*t*-test)

The translation initiation (mTOR signaling)/protein synthesis pathway is one of the key primary pathways positively regulated by MYC activation [28, 29]. Interestingly, this pathway was screened as one of the most significantly altered pathways by MP1 (Fig. 3). To validate this molecular mechanism(s) associated with MP1 anti-MB activity, we examined the expression/activation of key components translation (mTOR) signaling by western blotting in HD-MB03 and D-341 cell lines. We observed that MP1 treatment of MYC-amplified cells resulted in significantly downregulated expression of key phosphorylated/activated proteins (p-S6K, p-4EBP1 and p-eIF4E) of the translation pathway, in a dose-dependent manner, in both cell lines (Fig. 4A). These data suggest that MP1-induced inhibition of MYC suppresses the translation pathway.

Because of the key role of MYC/mTOR signaling in controlling global protein synthesis, we further validated whether the inhibition of MYC/mTOR represented a global blockage of protein synthesis. To this end, we performed a protein synthesis assay using a robust chemical method based on a cell permeable analogue of puromycin, O-Propargyl-puromycin (OPP), tagged with a fluorescent marker, in HD-MB03 and D-341 cell lines treated with MP1. In this assay, we used cycloheximide as the positive control for protein synthesis inhibition. This assay showed high protein synthesis activity in control solvent-treated cells and strong inhibition of protein synthesis by MP1 and cycloheximide in both cell lines (Fig. 4B), suggesting the anti-cancer potential of MP1 is partly based on targeting the MYC/mTOR-driven protein synthesis pathway.

#### MP1 targets energy metabolism in MYC-driven MB cells

Another major and immediate downstream effect of MYC activation is a dramatic increase in metabolism of the cells as it directly regulates and increases energy/ATP production rates through transcriptional and protein synthesis control to sustain the uncontrolled cancer cell proliferation [30–32]. Given such a prominent role of MYC in cancer metabolism, we next wanted to see whether MP1 modulates overall metabolism in a representative MYC-amplified HD-MB03 cell line. We performed targeted metabolomics using LC–MS/MS in control and MP1-treated HD-MB03 cells. In this metabolomics, we screened standard 173 metabolites encompassing all key cellular metabolic pathways. The metabolomics and pathway data analysis were performed using MetaboAnalyst 5.0 software. The primary metabolite profiles on control and MP1 treated conditions with three technical replicates that revealed distinct metabolic alterations as shown in our PCA plot (Supplementary Fig. S6). This plot represents the overall similarity/closeness among the technical replicates in both the conditions.

**Fig. 5:**
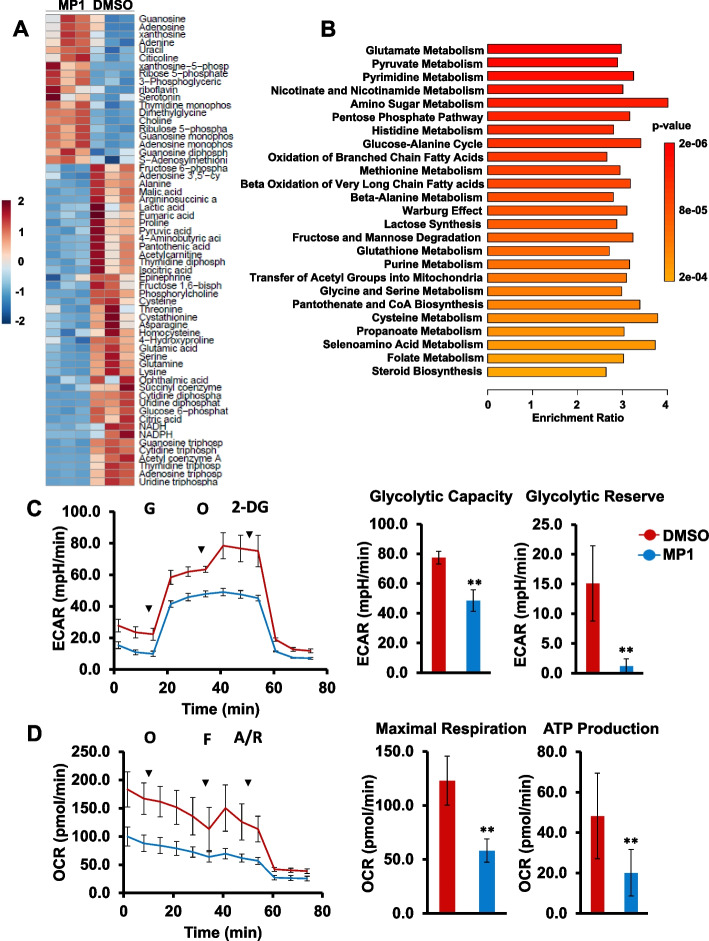
MP1 treatment alters energy metabolism in MB. **A** Heatmap showing top 60 metabolites altered in HD-MB03 cells treated with MP1 (0.25 µM) in triplicate for 24 h. Color intensity represents the magnitude of alteration in individual metabolites. Scale for color intensity is shown adjacent to the heatmap. **B** Pathway analysis showing significantly altered metabolic pathways in MP1 treated HD-MB03 cells, compared to DMSO solvent. Scale under the pathway plot shows the fold enrichment and color scale adjacent to pathway plot indicates significance (*p*-value) for altered pathways. **C** Extracellular acidification rate (ECAR) analysis for glycolytic activities in HD-MB03 cells after treatment with 0.25 µM MP1 for 24 h. G, glucose; O, oligomycin; 2-DG, 2-deoxyglucose. The bar graphs show glycolytic capacity and glycolytic reserve activities derived from ECAR activities shown in line graph. The results represent the mean ± SEM of three replicates. **p < 0.01 (Student t test, DMSO vs MP1). **D** Oxygen consumption rate (OCR) analysis for mitochondrial oxidative phosphorylation status in HD-MB03 cells after treatment with 0.25 µM MP1 for 24 h. O, oligomycin; F, FCCP; A/R, antimycin/rotenone. The bar graphs show maximal respiration and ATP production activities derived from OCR activities shown in line graph. The results represent the mean ± SEM of three replicates. **p < 0.01 (Student t test, DMSO vs MP1)

We next looked at the metabolites that showed statistically significant differences between the two conditions and performed student’s t-test with a *p*-value cutoff of 0.05 and found 60 metabolites to be altering significantly. A heatmap was generated to visualize the relative abundance of these metabolites in control (DMSO) and MP1 group samples (Fig. 5A). Overall, clear differences (60 potential primary metabolite) were observed between the control and the MP1, allow discrimination between the two groups. Using the list of altered metabolites, we performed pathway enrichment analysis and found that MP1 treatment led to significant changes in metabolites involved in 25 metabolic pathways (Fig. 5B). In MP1-treated cells there was a high frequency of alterations in energy related metabolic pathways such as amino acids (glutamate, histidine, methionine, glycine & serine, glutathione, and cysteine), glucose, pyruvate, amino sugar, pentose phosphate, Warburg effect, oxidation of fatty acids, and purine & pyrimidine metabolisms. Notably, these altered metabolic pathways are well known to be upregulated by MYC-activation, suggesting MP1 efficacy to target MYC-driven metabolism driven in MB cells.

Given the impact on energy metabolism, we further conducted key metabolic analyses to confirm whether the MP1 treatment affects glycolysis and oxidative phosphorylation in MYC-amplified MB (HD-MB03, D-341) cell lines. Using seahorse-based assays, we evaluated real-time glycolytic activity by measuring extracellular acidification rates (ECAR) and oxidative phosphorylation (OXPHOS) by measuring cellular oxygen consumption rates (OCRs) (Fig. 5C and D, Supplementary Fig. S7). In ECAR analysis, we observed that the MP1 treatment significantly reduced key indicators (glycolytic capacity, glycolytic reserve) of glycolysis, compared to control DMSO (Fig. 5C, Supplementary Fig. S7A). In OCR analysis, we found a significant reduction in mitochondrial maximal respiration rate (OCR) upon MP1 treatment, which was mirrored by the declined amount of OXPHOS-related ATP production (Fig. 5D, Supplementary Fig. S7B). Together, our results suggest that MP1 inhibits both the ECAR and OCR activities associated with energy metabolism, in MYC-driven MB cells.

#### MP1 shows synergy with mTOR inhibitor temsirolimus in vitro

**Fig. 6:**
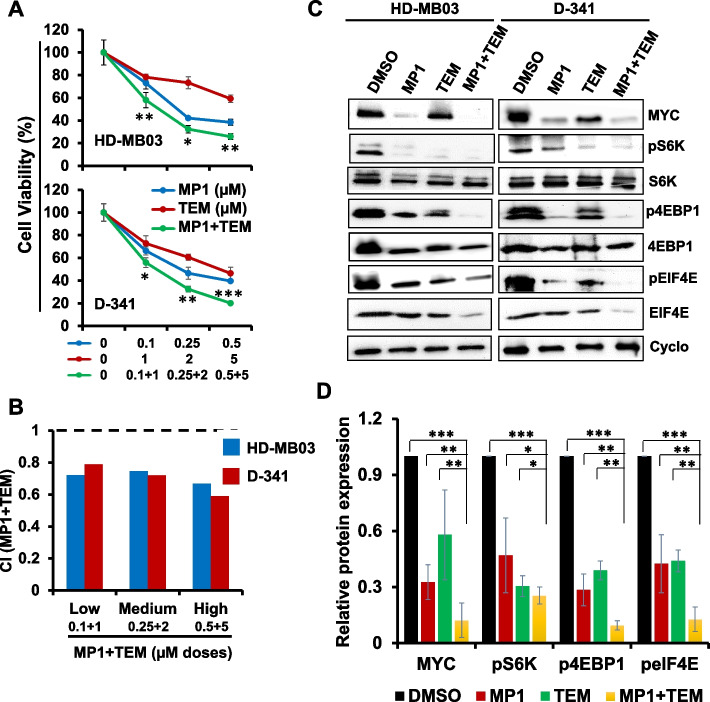
Synergistic effects of MP1 and temsirolimus (TEM) on MB cell growth. **A** Cell viability (MTT) assay showing dose-dependent growth effects of combined MP1-TEM in two MYC-amplified MB cell lines at 48 h. Viable cells (%) is relative to DMSO-treated cells. Values represent mean ± SEM. **B** Bar graphs show combination index (CI) analyses for the synergism of MP1 and TEM in MB cell lines. **C** Western blot images for the expression of key components of MYC and translation/mTOR signaling in two MYC-amplified MB cell lines following treatment with MP1 (0.25 µM) and TEM (2 µM) alone or combined for 24 h. Cyclophilin B (Cyclo) was used as the loading control in these analyses. **D** Bar graphs show the quantification of expression of key target proteins (shown in western blot images) relative to the DMSO control in the combined blots of HD-MB03 and D-341 cells after Cyclophillin B (loading control) normalization using ImageJ software. The values represent the mean ± SEM of three blot replicates. *p < 0.05; **p < 0.01; ***p < 0.001 (Student t test, vehicle/ or single agents vs. combination)

We next tested the efficacy of MP1 combined with temsirolimus (denoted as TEM in figures) to explore potential for synergistic growth inhibition of MB cells. Two MYC-amplified MB cell lines (HD-MB03, D-341) were treated with increasing concentrations of MP1 and temsirolimus, alone or in combination, for 48 h. As shown in Fig. 6A, co-treatment of MP1 and temsirolimus significantly suppressed growth of all MB cell lines in a dose-dependent manner, compared with single agent treatment. Combination index (CI) analyses by Chou-Talalay method [33] confirmed that the MP1/temsirolimus combination had strong synergistic inhibitory effects on MB cell growth, with CI values ranging 0.5—0.75 (Fig. 6B). This synergistic interaction was also confirmed using HSA & Loewe methods for synergy analysis (Supplementary Fig S8).

To establish the mechanism(s) associated with MP1/temsirolimus anti-MB activity, we examined the expression and activation of key components of MYC/mTOR signaling pathways by western blotting in MYC-amplified MB cell lines (Fig. 6C). Single agent treatments with MP1 or temsirolimus significantly suppressed expression of MYC and downregulated the levels of phosphorylated/activated signaling proteins (p-S6K, p-4EBP1, and p-eIF4E) of the mTOR (translation) pathway (Fig. 6D). Co-treatment with MP1 and temsirolimus further downregulated the expression of mTOR signaling components, as well as MYC expression, compared with single agent treatments. Together, these results suggested that MYC/mTOR signaling is a mechanistic target underlying the synergistic anti-MB potential of MP1 and temsirolimus.

### Combination of MP1 with temsirolimus reduces tumor growth in MYC-driven MB xenografts

**Fig. 7:**
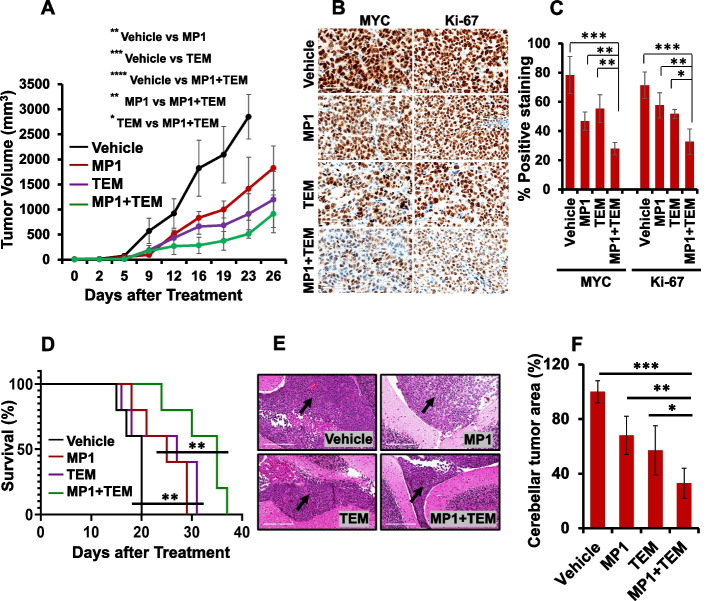
Combined in vivo effects of MP1 and temsirolimus (TEM) in subcutaneous and orthotopic MYC-MB bearing xenografts. **A** Tumor volume measurement of subcutaneously xenografted mice (*n* = 5 each group) following treatments. The differences between treatment groups represent ANOVA-based comparison of the tumor volumes 23 days post-treatment. *p < 0.05; **p < 0.02; ***p < 0.005; ****p < 0.001. **B** Representative IHC images (40 × magnification with 60 µm scale bar) of MYC and Ki-67 in treated subcutaneous xenografts. **C** The percentages of MYC and Ki-67 positive cells derived from histology scores were semi-quantified in the subcutaneous tumors of three xenografted mice following 21 days of treatment with inhibitors. *p < 0.05; **p < 0.01; ***p < 0.001 (ANOVA). **D** Survival analysis of orthotopic MYC-amplified MB-bearing mice (*n* = 5 each group) using Kaplan–Meier method. The survival comparisons between treatment groups (Vehicle vs. single agents or single agents vs. combination) were determined using the log-rank test. **E** Representative H&E images (20 × magnification with 200 µm scale bar) with an arrow bar showing the tumor growth in mouse cerebellum with each treatment. (F) Bar graph shows the quantification of H&E-stained tumor area in the cerebellum of each treatment group (*n* = 3) using Image-J. Tumor area in the treatment groups was calculated as percentages normalized to the tumor area in the control (vehicle treatment) group. *p < 0.05; **p < 0.01; ***p < 0.001 (ANOVA)

To evaluate the combined therapeutic potential of MP1 with temsirolimus against MYC-driven MB in vivo, NSG mice were xenografted subcutaneously with HD-MB03 cells and treated with vehicle control or MP1 and temsirolimus alone or the combination of MP1 with temsirolimus. As shown in Fig. 7A, treatment with MP1 and temsirolimus alone significantly suppressed tumor growth by approximately 50% to 75%, respectively, compared to vehicle control. Combination of MP1 with temsirolimus further significantly suppressed tumor growth by approximately 40% (compared to temsirolimus) and 60% (compared to MP1), suggesting the antitumor combination potential of MP1 with temsirolimus against MYC-driven MB in vivo. In addition, treatment with these inhibitors alone or combined did not cause significant changes in the total body weights and histopathology of vital organs between control and treatment groups (Supplementary Fig. S9), suggesting the tolerability of these therapies in mice.

We next determined the combined effect of inhibitors on the expression of MYC and Ki-67 (proliferation marker) in xenografted tumors. Immunohistochemical analysis showed that while MP1 and temsirolimus alone reduced the expression of MYC and Ki-67, the combination of MP1 and temsirolimus caused significant further reduction in the expression of MYC and Ki-67 in xenografted tumors (Fig. 7B and C). These data suggest that combinations not only reduced tumor growth, but also inhibited target proteins MYC and Ki-67 in subcutaneous xenografted tumors.

In addition to subcutaneous xenografts, we examined antitumor efficacy of combination therapy using orthotopic xenografts generated by intracerebellar injections of HD-MB03 cells into NSG mice. Ten days post tumor injection, mice (*n* = 5 per group) were treated with vehicle, MP1 or temsirolimus alone, or a combination of MP1/temsirolimus, similarly to the subcutaneous xenograft experiments. As shown in Fig. 7D, treatment with MP1 or temsirolimus alone significantly prolonged the survival of mice, compared to vehicle control. Combination of MP1 with temsirolimus further significantly prolonged survival, compared to MP1 or temsirolimus alone. In addition, we assessed tumor occurrence and growth in the cerebellum by histologic (H&E) analysis. Approximately 80% of the mice in treatment groups had significant tumor occurrence. We measured the tumor area in the cerebellum by Image-J and observed that co-treatment with MP1 or temsirolimus significantly reduced the tumor size in the cerebellum, compared with vehicle or MP1 or temsirolimus alone (Fig. 7F). There was also a significant reduction of tumor size with the treatment of MP1 or temsirolimus alone, compared to vehicle control (Fig. 7F). Together, in vivo results suggest a synergistic anti-MB potential of MP1/temsirolimus not only against subcutaneous tumors but also orthotopic tumors.

## Discussion

Despite intensive multimodal therapy, the prognosis for Group 3 MB patients with MYC-amplification remains extremely poor [5]. MYC has been one of the most appealing therapeutic targets for cancer drug development. Several approaches have attempted to inhibit MYC directly or indirectly at all levels of its regulation. However, despite substantial efforts, targeting MYC with clinical grade small molecules still represents an intractable challenge, particularly at the protein level. The fact that important functional domains of MYC protein are intrinsically disordered and lack an enzymatically active site, poses an enduring obstacle for the design of effective MYC inhibitors [11, 12]. In this study, we report a novel marinopyrrole-derived agent, MP1 that shows desirable anti-MYC and anti-cancer activities in MYC-driven MB, both in vitro and in vivo. This study provides the first preclinical evidence of targeting MYC with marinopyrrole MP1 against most aggressive MB.

In addition to antibacterial activities, marinopyrroles have previously proven to have anticancer activities by binding to and destabilizing MCL-1, an anti-apoptotic protein [15, 16]. Our recent study in MYCN-amplified neuroblastoma revealed that MP1 also targets MYCN (a MYC homologue) along with MCL-1, leading to autophagy stimulation and energy metabolism inhibition [19]. MYC itself is a direct regulator of MCL-1 transcription and there is often tumorigenic co-regulation of MYC and MCL-1 proteins [34–36]. While MYCN inhibition by MP1 is described, inhibition of MYC itself has not been reported. In the present study, we provide more mechanistic rationale for MYC targeting by MP1. Our findings demonstrate that MP1 not only inhibits MYC protein expression in MB, but also key pathways under its transcriptional control, such as cell cycle, protein synthesis/translation and metabolic pathways. Importantly, we and others have previously shown that mTOR/translation and metabolic pathways are highly activated in MYC-driven MB [23, 37], which is consistent with MP1 having anticancer mechanism(s) that target these cellular pathways downstream of MYC. Together, these data suggest that MYC is the key target for MP1. Whether this is based on direct binding to MYC, or an indirect effect is an active area of investigation.

In this study, we observed superior responsiveness of MYC-amplified MB cell lines to MP1, compared to non-MYC-amplified MB cell lines. We have confirmed this dependency using induced MYC overexpression in a non-MYC-amplified/low-MYC cell line model. We observed minimal responsiveness of the non-MYC-amplified (Daoy, ONS-76) MB cell lines to MP1. The fact that these lines have not shown completely overwhelmed with MYC expression [38] and may not represent the full spectrum of non-MYC-amplified MB. Therefore, we also see some efficacy of MP1 in these lines with low-MYC expression. Together, these data suggest MYC-dependent specificity of MP1 in MB.

Among the top 50 genes most significantly downregulated by MP1, we identified *PROM1* (*CD133,* neural stem cell) as one of the most cancer relevant genes, particularly considering its crucial roles in neurodevelopment and Group 3 MB tumorigenesis [27, 39–42]. Therefore, as a proof of concept, we further validated the reduced expression of CD133 at both mRNA and protein levels in MYC-amplified MB cell lines using RT-PCR and western blotting. We therefore hypothesize that reduced expression of CD133 is mechanistically important, along with suppressed MYC expression, in the anticancer effect of MP1 in MB. Further investigation is required to further understand the anticancer mechanism(s) of MP1 targeting the MYC-CD133 axis in MB. Among the top downregulated 50 genes, there were other cancer relevant genes such as cell cycle associated (*CCNF1, CCNB1, ZWINT*), cancer signaling (*STK32B, IGF1, FLT1*) and splicing associated (*HNRNPM, HNRNPU, SRSF1*) genes, which are active areas to investigate further. We have not pursued studies investigating further on upregulated genes/pathways upregulated by MP1 as these appeared not to be directly relevant to cancers, particularly in brain cancer aspects.

In a search of potential drug targets that can be combined with MP1, we analyzed the regulation of the MYC-target or its associated gene sets/pathways upon treatment of the MYC-amplified MB (HD-MB03) cells with MP1. Screening for top gene sets/pathways suppressed by MP1, we identified translation (mTOR)/protein synthesis pathways as one of the most drug targetable pathways. Therefore, we chose the mTOR/translation pathway as an additional target to explore further in combination with MP1-directed MYC inhibition. Specifically, we used temsirolimus (an FDA approved clinical drug) to target the mTOR/translation pathway in combination with MP1. Our results suggest that MP1 also targets energy metabolism, a downstream cellular mechanism of both MYC and mTOR signaling [30, 43], providing additional rationale why combining of MYC and mTOR inhibitors could be effective against MYC-driven MB. Expectedly, our combination data confirm the anticancer synergy of targeting these two pathways in MYC-driven MB.

The dosing and treatment schedule for MP1 in this study was based on our previous studies [19, 20]. In these studies, we have identified MP1 as an orally bioavailable compound with favorable PK/PD properties and stability in mice. Particularly, biodistribution profiles in these studies suggest that MP1 crosses the blood–brain barrier [19]. The brain concentrations of MP1 in mice were in the ranges of its IC_50_s we observed in cell lines, suggesting MP1 can reach to and target tumors in the brain. In addition, MP1 appeared to be well tolerated at a 15 mg/kg dose (half of 30 mg/kg MTD), with no obvious toxicity [20]. Together, these data support MP1 as a potential agent for MB therapeutics.

Ongoing work, however, is required to determine optimal strategies to incorporate MP1 into frontline therapy for children with MYC-amplified MB. Based on our data, assessing the efficacy of MP1 in combination with metabolic inhibitors is warranted. Furthermore, studies in cancers have also identified MYC inhibition as a radiosensitization or chemosensitization mechanism [44, 45] and it should be explored in MB if MP1-induced MYC inhibition is a promising option to be combined with treatment modalities such as chemotherapy and radiotherapy. Our findings also highlight the value of further investigation on combining MP1 with temsirolimus using patient-derived orthotopic xenografts, in order to take this approach to clinical investigation. Experimental parameters to test our findings using patient-derived xenografts in a MB orthotopic model are currently under development in our lab.

## Conclusions

In summary, our results demonstrate that MP1 inhibits MYC protein expression, MYC-targeted transcription, and its regulated key tumorigenic programs such as translation/mTOR and energy metabolism pathways, leading to suppressed proliferation/survival of MYC-driven MB cells. In addition, MP1 shows synergistic anti-cancer efficacies with temsirolimus in MYC-driven MB cells by targeting the mTOR signaling pathway. Our in vivo results further confirm this synergy in reducing tumor growth and prolonging survival in subcutaneously or orthotopically MYC-driven MB bearing mice. Thus, our findings highlight the targeting of MYC and its regulated pathways by which MP1 modulates proliferation/survival and exerts its anti-MB activity, warranting further preclinical and clinical investigations to take this approach to clinical setting. Since MYC is a common oncogene in a wide variety of cancers, the strategy of MYC inhibition by MP1 might also be useful in other MYC-addicted cancers.

### Supplementary Information

**Additional file 1: Supplementary Table 1.** The top 50 genes most significantly (*p*<0.0001) downregulated by the MP1 in HD-MB03 cells. **Supplementary Figure S1.** Quantification for the expression of key target proteins. **Supplementary Figure S2.** MP1 modulates target gene sets. **Supplementary Figure S3.** Top pathways modulated by MP1. **Supplementary Figure S4.** Effects of MP1 on* PROM1* (*CD133*) and *MYC* mRNA expression. **Supplementary Figure S5.** Effect of MP1 on the stability of MYC protein. **Supplementary Figure S6.** Principal Component Analysis (PCA) between DMSO and MP1 treatment groups. **Supplementary Figure S7.** MP1 treatment alters energy metabolism in MB. **Supplementary Figure S8.** Synergy analysis between MP1 and TEM in MYC-amplified MB cells. **Supplementary Figure S9.** Effects of inhibitors on body weight and histology of the MB xenograft mice.

## Data Availability

All data generated or analyzed in this study are included in this manuscript except RNA sequencing raw data, which is available from the corresponding author on reasonable request.

## Abbreviations

CI: Combination index
DMSO: Dimethyl-sulfoxide
FBS: Fetal bovine serum
GSEA: Gene set enrichment analysis
h: Hour
MB: Medullooblastoma
MTT: 3-(4,5-Dimethylthiazol-2-yl)-2,5-diphenyltetrazolium bromide
MP1: Marinopyrrole/Pyrrolomycin-1
MYC: V-myc avian mylocytomatosis viral oncogene homolog
MYCN: V-myc avian mylocytomatosis viral oncogene neuroblastoma homolog
nM: Nanomolar
qRT-PCR: Quantitative real-time polymerase chain reaction
RNA-seq: RNA sequencing
TEM: Temsirolimus
µM: Micromolar
