# Supplementary material for: Marinopyrrole derivative MP1 as a novel anti-cancer agent in group 3 MYC-amplified Medulloblastoma
Source: J Exp Clin Cancer Res. 2024 Jan 11;43:18. doi: 10.1186/s13046-024-02944-w (PMC10782703; doi:10.1186/s13046-024-02944-w)
Supplement: Supplementary file 1 — Additional file 1: Supplementary Table 1. The top 50 genes most significantly (p<0.0001) downregulated by the MP1 in HD-MB03 cells. Supplementary Figure S1. Quantification for the expression of key target proteins. Supplementary Figure S2. MP1 modulates target gene sets. Supplementary Figure S3. Top pathways modulated by MP1. Supplementary Figure S4. Effects of MP1 on PROM1 (CD133) and MYC mRNA expression. Supplementary Figure S5. Effect of MP1 on the stability of MYC protein. Supplementary Figure S6. Principal Component Analysis (PCA) between DMSO and MP1 treatment groups. Supplementary Figure S7. MP1 treatment alters energy metabolism in MB. Supplementary Figure S8. Synergy analysis between MP1 and TEM in MYC-amplified MB cells. Supplementary Figure S9. Effects of inhibitors on body weight and histology of the MB xenograft mice. [file 13046_2024_2944_MOESM1_ESM.docx]

**Supplementary Data**

| **Gene Name** | Fold-Change | **Gene Name** | Fold-Change |
| --- | --- | --- | --- |
| **BPIFB4** | -38.4996 | **CTSV** | -6.30966 |
| **DHX9** | -2.67578 | **FABP5** | -4.01937 |
| **NEDD9** | -4.01277 | **FUS** | -3.03689 |
| **GBA** | -4.40083 | **TPM3** | -2.64384 |
| **SUSD2** | -16.5805 | **RBM12** | -2.82823 |
| **CDH11** | -4.00537 | **TPGS2** | -4.06916 |
| **AHR** | -4.19542 | **PROM1** | -2.75546 |
| **EFHD2** | -3.68856 | **FEN1** | -3.10549 |
| **CCNF** | -3.78839 | **FAM83D** | -3.56408 |
| **ANK3** | -7.26046 | **CRABP2** | -30.4179 |
| **NCLN** | -2.7353 | **PCSK5** | -11.7096 |
| **KIAA0101** | -3.81241 | **GRIK3** | -13.9107 |
| **STK32B** | -56.9934 | **RBM14** | -5.19286 |
| **LAMP1** | -2.90801 |  |  |
| **CCNB1** | -3.4855 |  |  |
| **ACTN1** | -2.70821 |  |  |
| **TEX261** | -3.54522 |  |  |
| **CTSA** | -3.85071 |  |  |
| **IGF1** | -32.6951 |  |  |
| **MYL9** | -5.3993 |  |  |
| **HNRNPU** | -2.91857 |  |  |
| **ZWINT** | -3.10472 |  |  |
| **HNRNPM** | -4.44843 |  |  |
| **CDC20** | -4.22088 |  |  |
| **PSMD1** | -2.73866 |  |  |
| **L1TD1** | -32.0954 |  |  |
| **TPX2** | -2.49614 |  |  |
| **GALNT2** | -2.80295 |  |  |
| **PGAM1** | -3.25405 |  |  |
| **SFPQ** | -3.65934 |  |  |
| **NSUN2** | -2.73474 |  |  |
| **SRSF1** | -2.24914 |  |  |
| **FLT1** | -5.99943 |  |  |
| **CDH6** | -10.6514 |  |  |
| **KPNA2** | -2.89558 |  |  |
| **MYBL2** | -3.70842 |  |  |

**Supplementary Table 1**. The top 50 genes most significantly (p<0.0001) downregulated by the MP1 in HD-MB03 cells.


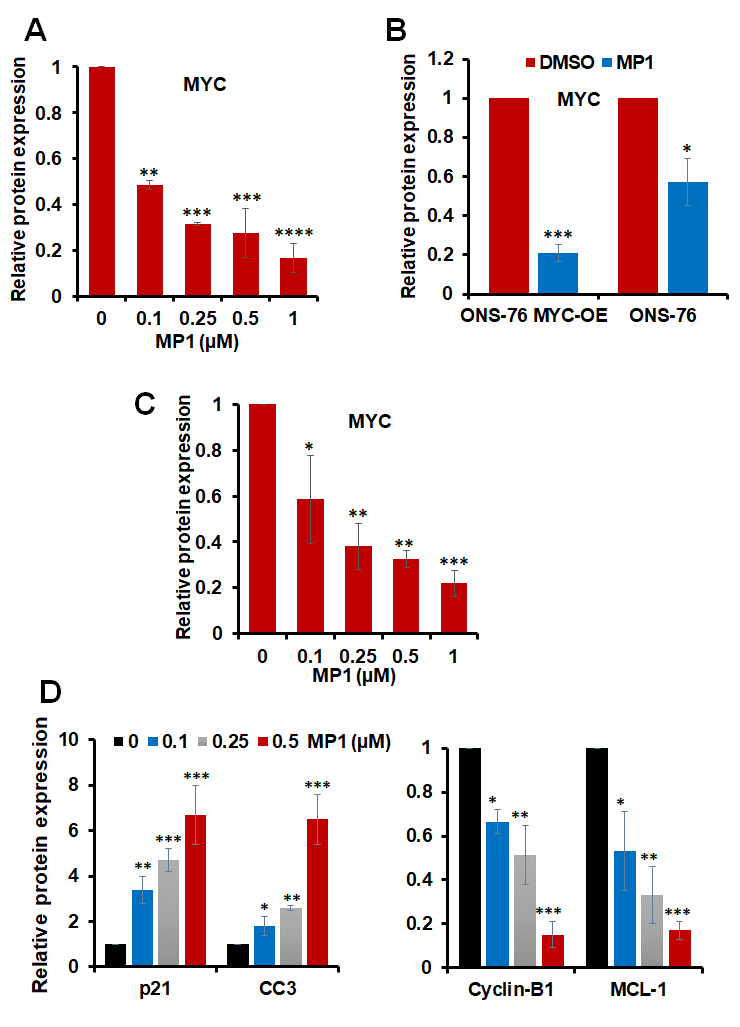


**Supplementary Figure S1. Quantification for the expression of key target proteins.** Bar graphs A, B, C, and D show the expression of indicated proteins for the western blot images shown in main Figures 1B, 1D, 1E, and 2D, respectively, relative to the DMSO control in the combined blots of HD-MB03 and D-341 cell lines after loading control normalization using ImageJ software. The values represent the mean ± SEM of three blot replicates. *p<0.05; **p<0.01; ***p<0.001.


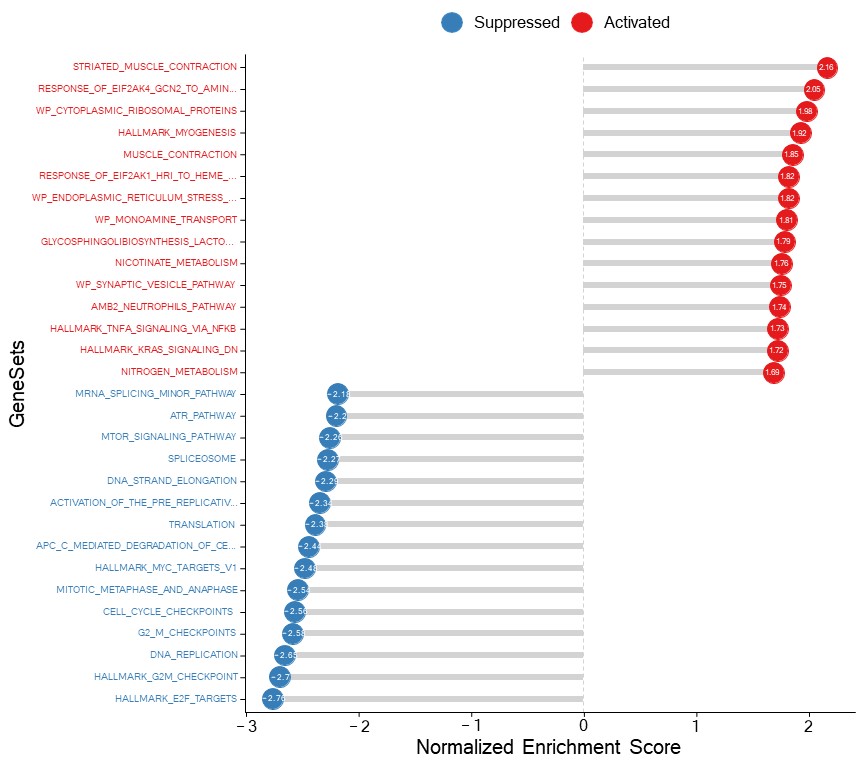


**Supplementary Figure S2.** **MP1 modulates target gene sets.** GSE analysis (GSEA) was performed using RNA-sequencing based differential gene expression in HD-MB03 cells 24 h after treatment with control (DMSO) solvent and MP1 (0.25 µM). GSEA generated top enriched gene sets/pathways, revealing modulation of MYC, translation/mTOR, splicing and cell cycle target gene sets by MP1. These pathways are represented with p<0.01 and FDR<0.25 cut-off.


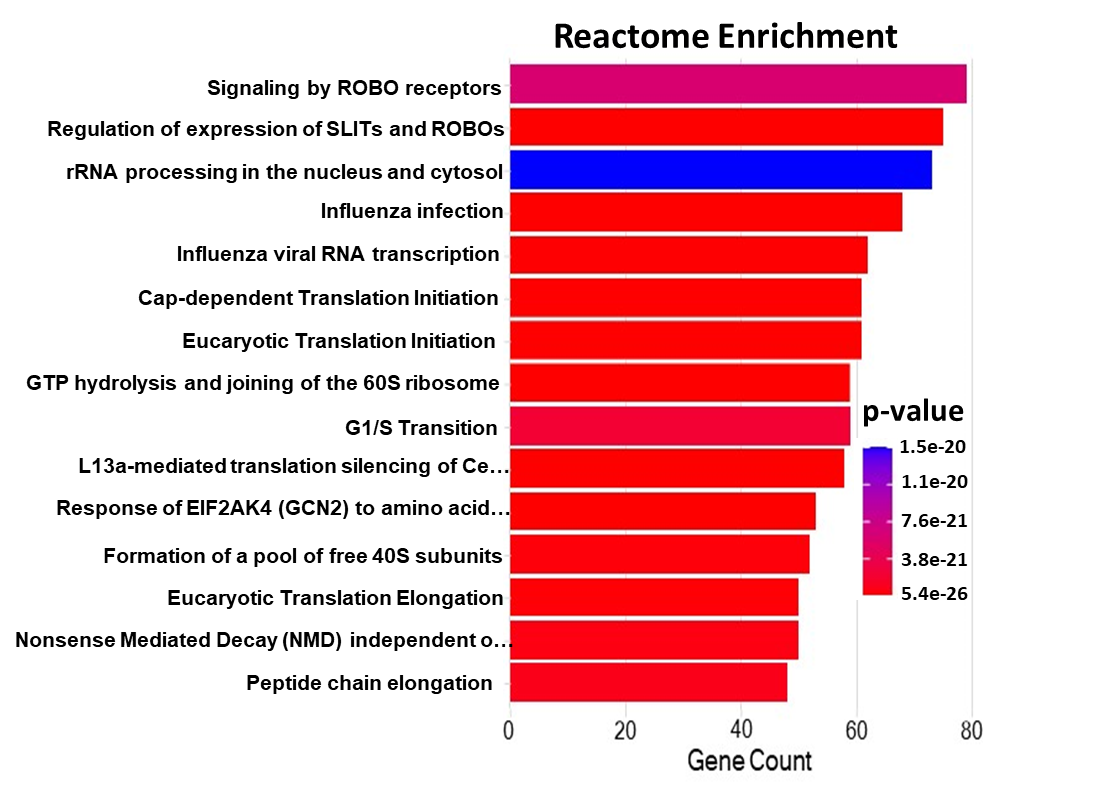


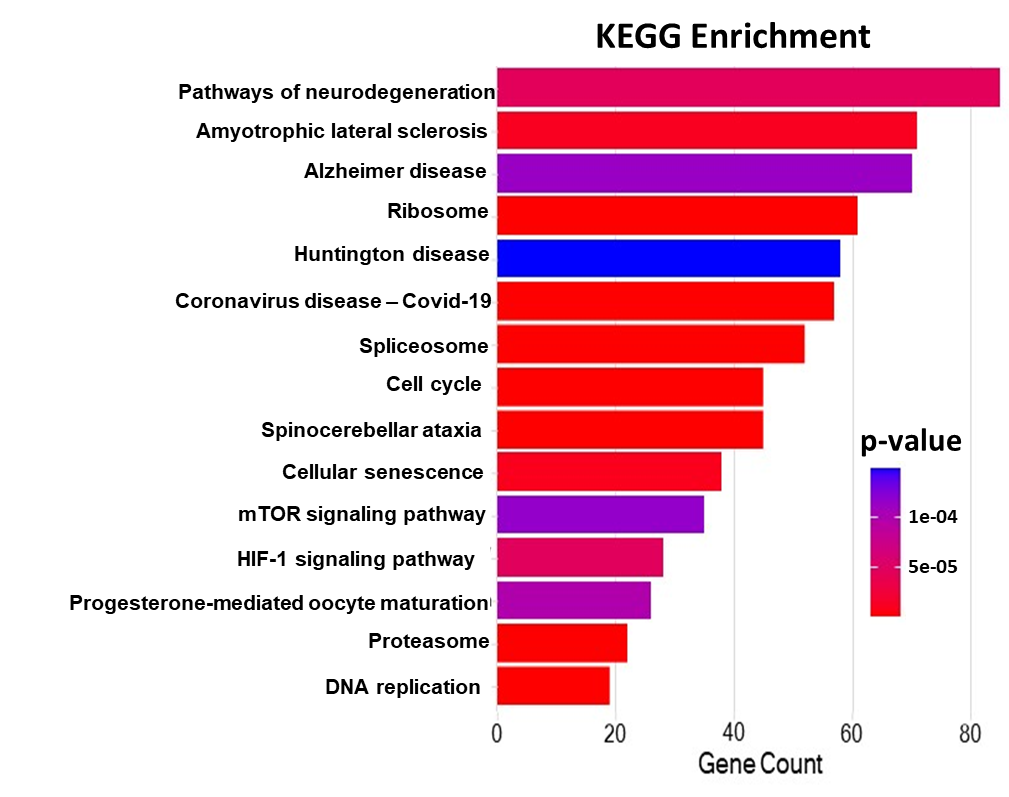


**Supplementary Figure S3.** **Top pathways modulated by MP1.** KEGG and Reactome pathway enrichment analyses were performed using RNA-sequencing based differential gene expression in HD-MB03 cells 24 h after treatment with control (DMSO) solvent and MP1 (0.25 µM). Top 15 pathways generated by these analyses, confirming modulation of MYC-associated pathways, particularly translation/mTOR, cell cycle and splicing pathways by MP1.


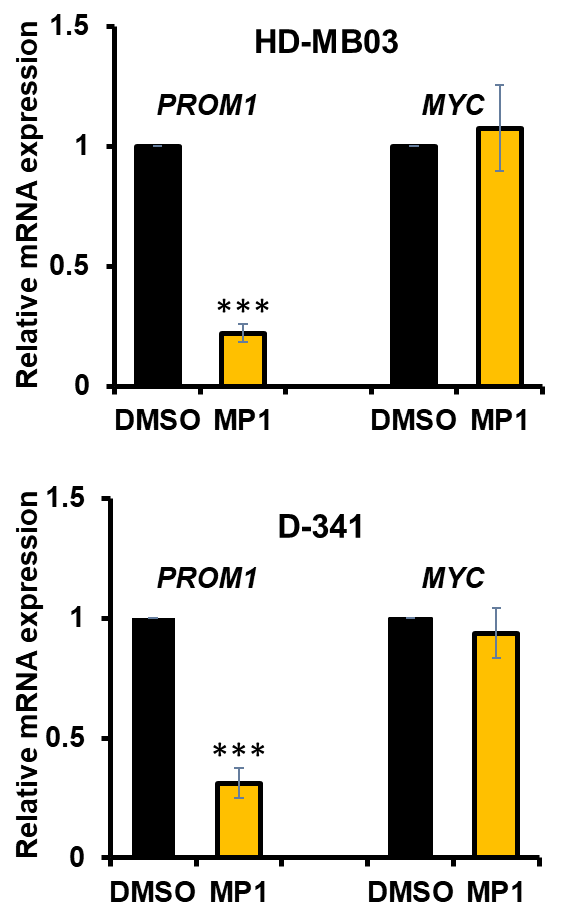


**Supplementary Figure S4. Effects of MP1 on *PROM1* (*CD133*) and *MYC* mRNA expression.** Quantitative RT-PCR showing the mRNA levels of *PROM1* and *MYC* in two MYC-amplified cell lines (HD-MB03, D-341) treated with 0.25 µM MP1 for 24 h. MP1 vs DMSO solvent (*******p<0.001, Student-t-test).


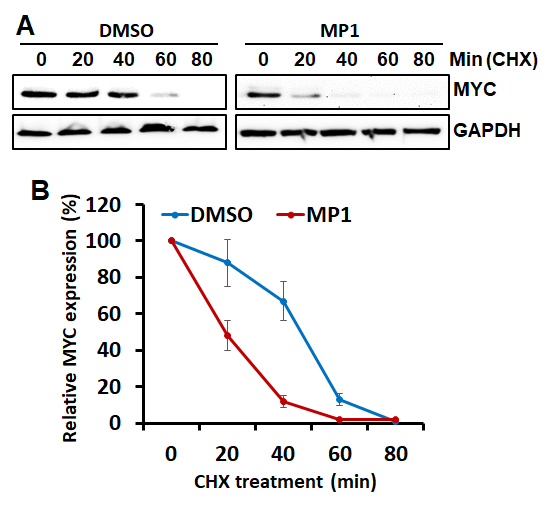


**Supplementary Figure S5. Effect of MP1 on the stability of MYC protein.** (A) Western blot analysis of MYC expression after 50 μg/ml CHX treatment following treatment of control solvent (DMSO) and MP1 (0.25 µM for 24 h) in HD-MB03 cells. (B) Densitometric quantification of MYC protein expression using three replicates of western blot image shown in “A”.


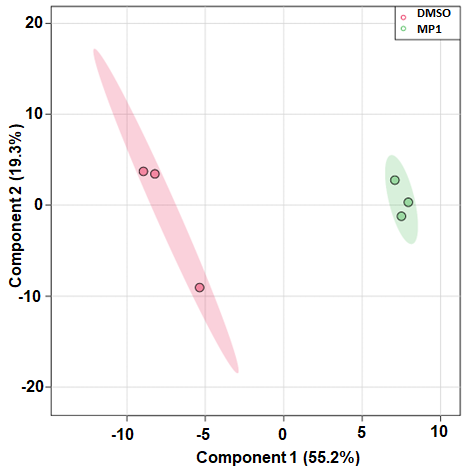


**Supplementary Figure S6. Principal Component Analysis (PCA) between DMSO and MP1 treatment groups.** PCA plot showing the segregation of vehicle (DMSO) and MP1 (0.25 µM for 24 h) treated HD-MB03 cells based on their metabolite profiles. Each colored circle represents a biological replicate of the treatment condition. Component 1 indicates the degree of variation between the groups based on their total metabolite content, and component 2 indicates the differences within the groups.


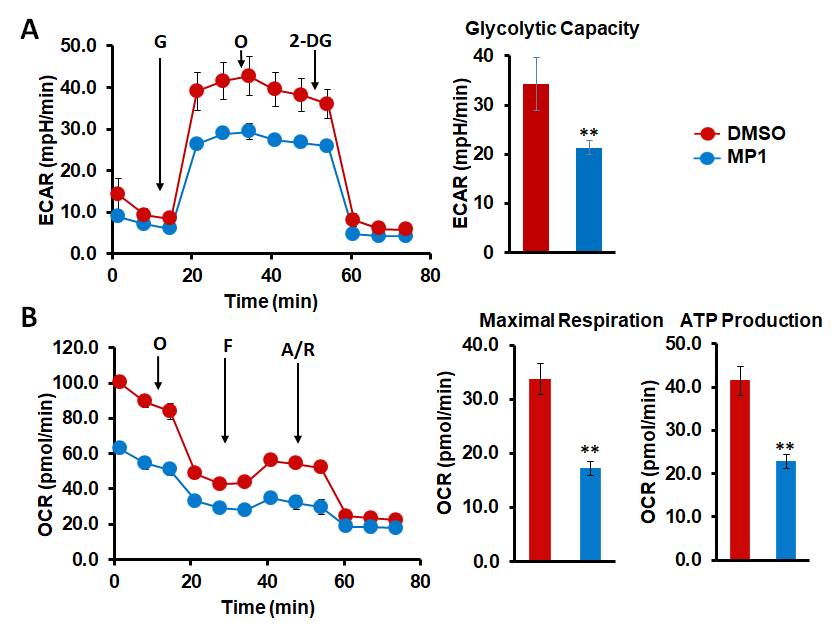


**Supplementary Figure S7. MP1 treatment alters energy metabolism in MB. (A)** Extracellular acidification rate (ECAR) analysis for glycolytic activities in D-341 cells after treatment with 0.25 µM MP1 for 24 h. G, glucose; O, oligomycin; 2-DG, 2-deoxyglucose. The bar graphs show glycolytic capacity derived from ECAR activities shown in line graph. The results represent the mean ± SEM of three replicates. **p<0.01 (Student t test, DMSO vs MP1). **(B)** Oxygen consumption rate (OCR) analysis for mitochondrial oxidative phosphorylation status in D-341 cells after treatment with 0.25 µM MP1 for 24 h. O, oligomycin; F, FCCP; A/R, antimycin/rotenone. The bar graphs show maximal respiration and ATP production activities derived from OCR activities shown in line graph. The results represent the mean ± SEM of three replicates. **p<0.01 (Student t test, DMSO vs MP1).


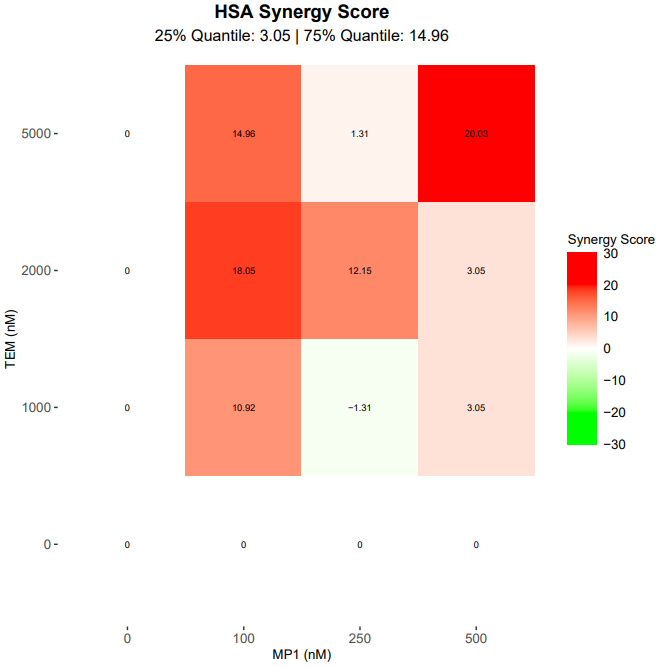

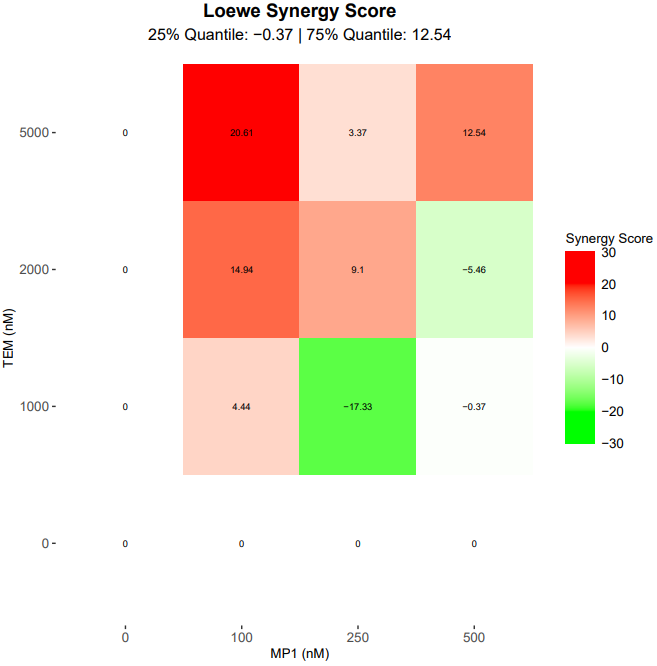


**Supplementary Figure S8. Synergy analysis between MP1 and TEM in MYC-amplified MB cells.** Heatmaps (generated from HSA and Loewe methods) showing the synergy scores of MP1 and TEM treated HD-MB03 cells in a dose-dependent manner. The synergy score indicates the degree of synergy between the drugs. > -10 score is generally considered as the synergistic. These synergy were analyzed using a Bioconductor synergy finder package ( [https://bioconductor.org/packages/release/bioc/vignettes/synergyfinder/inst/doc/Usertutorual_of_the_SynergyFinder_plus.html [bioconductor.org](https://bioconductor.org/packages/release/bioc/vignettes/synergyfinder/inst/doc/Usertutorual_of_the_SynergyFinder_plus.html%20%5bbioconductor.org%5d)).

**A**


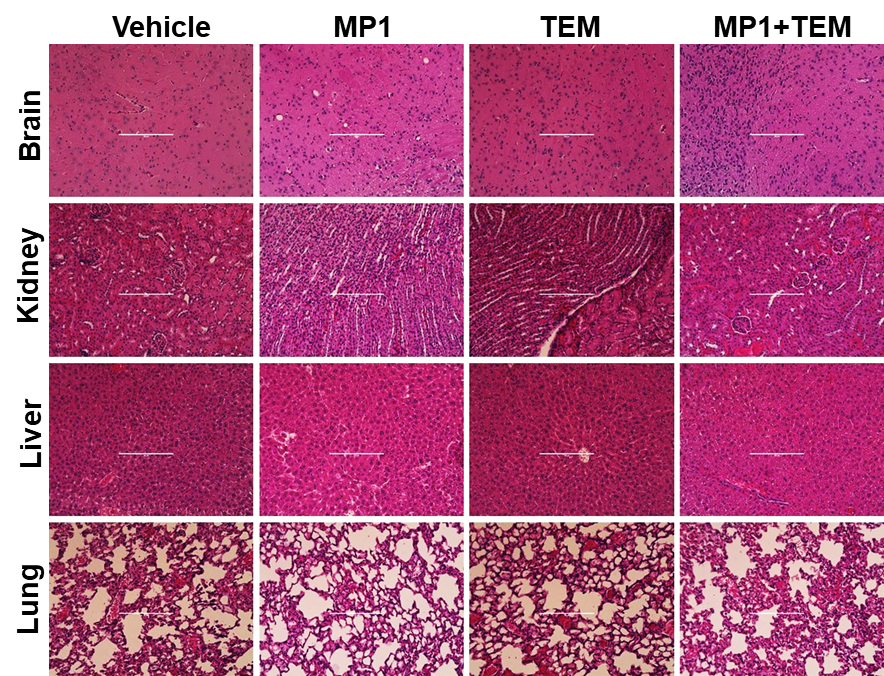


**B**

**Supplementary Figure S9. Effects of inhibitors on body weight and histology of the MB xenograft mice.** **(a)** The line graph is Showing the mean body weight of mice following treatment with inhibitors alone or combined as indicated. **(b)** Histopathology (H&E) of the vital organs of MB xenografts following 21 days post treatment with inhibitors. The images were scanned and captured using digital scanner EVOS Image system at 20x magnification.
